# Concomitant high expression of ERα36, EGFR and HER2 is associated with aggressive behaviors of papillary thyroid carcinomas

**DOI:** 10.1038/s41598-017-12478-1

**Published:** 2017-09-25

**Authors:** Yu-Jie Dai, Yi-Bo Qiu, Rong Jiang, Man Xu, Le Zhao, George G. Chen, Zhi-Min Liu

**Affiliations:** 1 Department of Biochemistry and Molecular Biology, Molecular Medicine and Cancer Research Center, Chongqing Medical University, Chongqing, China; 2 Department of Pathology, Molecular Medicine and Cancer Research Center, Chongqing Medical University, Chongqing, China; 3 Department of Surgery, The Chinese University of Hong Kong, Prince of Wales Hospital, Shatin, Hong Kong China

## Abstract

ERα, ERβ, PR, ERα36, EGFR and HER2 mRNA and protein expression in papillary thyroid carcinoma (PTC) were examined by real time RT-PCR and immunohistochemical staining. The mRNA and protein expression of ERα and PR were gradually increased and those of ERβ were gradually decreased from normal thyroid tissues to nodular hyperplasias (P < 0.05) and to PTCs (P < 0.05). However, the mRNA and protein expression of ERα36, EGFR and HER2 were only significantly increased in PTCs when compared with those in normal thyroid tissues (P < 0.001) and nodular hyperplasias (P < 0.001). There was some correlation between ERα, ERβ and PR, and between ERα36, EGFR and HER2 protein expression in PTCs. As for ERα, ERβ and PR, there was a significant positive correlation between ERα and PR, and a significant negative correlation between ERα and ERβ and between PR and ERβ protein expression. As for ERα36, EGFR and HER2, there was a significant positive correlation between ERα36, EGFR and HER2 protein expression in PTCs. Concomitant high expression of ERα36, EGFR and HER2 was strongly associated with aggressive behaviors including extrathyroidal extension (ETE), lymph node metastasis (LNM) and high TNM stage in PTCs (P < 0.001).

## Introduction

Clinical and epidemiological studies have shown that papillary thyroid carcinoma (PTC) accounts for 80% of thyroid malignancy and is three times more common in women than in men, with the greatest gender difference observed during reproductive age and the decreased incidence after menopause^1,2^. The elevated risk was reported in women who used estrogen for gynecological problems^3,4^. These data strongly suggest that estrogen may be involved in the occurrence and development of PTC, as largely demonstrated in breast, endometrial and ovarian carcinomas^5^.

It is well known that estrogen manifests its physiologic and pathophysiologic actions through its interaction with two estrogen receptors, ERα and ERβ, which belong to the nuclear steroid hormone receptor family and function as hormone-dependent transcription factors to induce transactivation of a series of estrogen-dependent target genes^6,7^. Progesterone receptor (PR) is a paragon of estrogen-induced protein and is employed as a biomarker of ERα function and breast cancer prognosis^8–10^. However, there is increasing evidence demonstrating that estrogen also acts via plasma membrane receptor(s). One of the key candidates for the membrane mediated action of estrogen is a novel 36 kDa variant of full-length (66 kDa) ERα and is designated as ERα36. Compared with ERα, ERα36 retains DNA-binding, partial dimerization and ligand-binding domains but lacks both transcriptional activation domains (AF-1 and AF-2)^11,12^. The C-terminal 27-amino acid domain of ERα36 is unique and takes the place of the last 138 amino acids encoded by exon 7 and 8 of the ERα gene. This unique sequence may broaden the ligand-binding spectrum of ERα36 so that it is able to bind more ligands. In addition to estrogen, ERα36 also binds tamoxifen and fulvestrant, two ER inhibitors widely used in the clinic, which creates an agonistic response and is involved in resistance to classical endocrine therapy in estrogen-related cancers^13,14^. ERα36 mainly locates in the cytoplasm, as well as on the cell surface where it mediates non-genomic estrogen signaling through cross-talk with growth factor receptors and other signaling molecules (such as MAPK/ERK, PI3K/AKT and PKC) and promotes cell growth, invasion, migration and resistance to endocrine therapy^13–17^. High expression of ERα36 has been observed in breast cancer stem cells^18–21^, ER-positive and -negative human breast carcinomas^22^, endometrial carcinomas^23^ and gastric carcinomas^24^, which has shown to be associated with malignancy, invasion, metastasis, resistance to treatment and poor prognosis in these types of carcinomas. However, so far, no study dealt with ERα36 expression and its correlation with clinicopathological features of PTC.

Epidermal growth factor receptor (EGFR) and its family members are a group of receptor tyrosine kinases on cell surface. Signaling occurs through both homo- and hetero-dimerization between members of the family, which induce cell proliferation, motility and invasion^25^. Interestingly, the preferred dimer partner of EGFR is the family member HER2 (also called EGFR2)^26^. The overexpression of EGFR and HER2 is frequently found in a variety of human malignancies such as breast^27^, endometrial^28^, colon^29^, gastric^30^ and papillary thyroid carcinomas^31,32^, and high expression of EGFR and HER2 occurs at an advanced stage of malignancy characterized by metastatic competence and poor prognosis.

In this study, we will simultaneously examine ERα, ERβ, PR, ERα36, EGFR and HER2 expression in PTCs, nodular hyperplasias and normal thyroid tissues by using real-time RT-PCR and immunohistochemical staining, systematically assess the association of their expression with clinicopathological features and evaluate the potential usefulness of these molecules in prediction for aggressive behaviors of PTCs.

## Results

### mRNA expression of ERα, ERβ, PR, ERα36, EGFR and HER2 in PTCs, nodular hyperplasias and normal thyroid tissues

To compare gene expression of ERα, ERβ, PR, ERα36, EGFR and HER2 in PTCs, nodular hyperplasias and normal thyroid tissues, 10 PTCs, 10 nodular hyperplasias and 10 normal thyroid tissues were collected to detect the mRNA levels of these molecules using real-time RT-PCR. As shown in Table 1, ERα, ERβ and PR mRNA were expressed in PTCs, nodular hyperplasias and normal thyroid tissues. ERα and PR mRNA levels were significantly higher in PTCs than in nodular hyperplasias (P < 0.001 for both ERα and PR) and normal thyroid tissues (P < 0.001 for both ERα and PR). However, ERβ mRNA level was significantly lower in PTCs than in nodular hyperplasias (P < 0.001) and normal thyroid tissues (P < 0.001). Furthermore, nodular hyperplasias had higher ERα and PR and lower ERβ mRNA levels when compared with normal thyroid tissues (P < 0.001 for ERα, ERβ and PR). These results indicated that ERα and PR mRNA levels were gradually increased and ERβ mRNA level was gradually decreased from normal thyroid tissues to nodular hyperplasias and to PTCs. As for ERα36, EGFR and HER2, the mRNA levels of the three molecules were significantly higher in PTCs than in nodular hyperplasias (P < 0.001 for ERα36, EGFR and HER2) and normal thyroid tissues (P < 0.001 for ERα36, EGFR and HER2). There were no statistically significant differences in mRNA levels of ERα36, EGFR and HER2 between nodular hyperplasias and normal thyroid tissues (P = 0.137, 0.117, 0.157 for ERα36, EGFR and HER2, respectively). These results indicated that increased mRNA expression of ERα36, EGFR and HER2 was only associated with the occurrence of PTC, but not with that of nodular hyperplasia.

**Table 1:** mRNA expression of ERα, ERβ, PR, ERα36, EGFR and HER2 in PTCs, nodular hyperplasias and normal thyroid tissues.

Groups(n = 10)	ERα (ΔCT, *P* value)	ERβ (ΔCT, P value)	PR (ΔCT, P value)	ERα36 (ΔCT, *P* value)	EGFR (ΔCT, P value)	HER2 (ΔCT, P value)
Normal thyroid tissues	4.15 ± 0.82	28.37 ± 4.87	4.46 ± 0.95	1.50 ± 0.28	1.78 ± 0.47	1.93 ± 0.43
Nodular hyperplasias	15.53 ± 3.95(<0.001^a^)	18.21 ± 4.92(<0.001^a^)	16.54 ± 5.20(<0.001^a^)	1.73 ± 0.35(0.137^a^)	2.15 ± 0.52(0.117^a^)	2.29 ± 0.64(0.157^a^)
PTCs	28.72 ± 5.16(<0.001^b^)	8.85 ± 2.13(<0.001^b^)	29.26 ± 8.21(<0.001^b^)	32.39 ± 6.65(<0.001^b^)	36.52 ± 7.01(<0.001^b^)	38.40 ± 7.13(<0.001^b^)
	(<0.001^c^)	(<0.001^c^)	(0.001^c^)	(<0.001^c^)	(<0.001^c^)	(<0.001^c^)

Mean ± SD of ERα, ERβ, PR, ERα36, EGFR and HER2 mRNA expression in PTCs, nodular hyperplasias and normal thyroid tissues after normalization to GAPDH. P-values derived using Mann-Whitney U test; ^a^Stands for significant difference between normal thyroid tissues and nodular hyperplasias; ^b^Stands for significant difference between PTCs and normal thyroid tissues; ^c^Stands for significant difference between PTCs and nodular hyperplasias; P < 0.05 was considered to be statistically significant.

### Immunohistochemical expression of ERα, ERβ, PR, ERα36, EGFR and HER2 in PTCs, nodular hyperplasias and normal thyroid tissues

**Figure 1:**
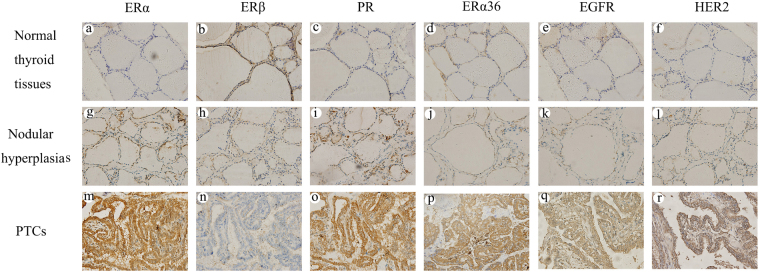
Immunohistochemical staining for ERα, ERβ, PR, ERα36, EGFR and HER2. Columns correspond to immunostaining for ERα, ERβ, PR, ERα36, EGFR and HER2, respectively. The first row (**a**–**f**) shows the representatives of immunostaining for normal thyroid tissues. The second row (**g**–**l**) displays the representatives of immunostaining for nodular hyperplasias. The third row (**m**–**r**) shows the representatives of immunostaining for PTCs. All the pictures are in high-power fields (×400).

ERα, ERβ, PR, ERα36, EGFR and HER2 protein expression were examined by immunohistochemical staining and the representatives of immunostaining for the six molecules were illustrated in Fig. 1. As shown in Fig. 1, ERα, ERβ and PR protein were expressed in PTCs, nodular hyperplasias and normal thyroid tissues. Compared with normal thyroid tissues and nodular hyperplasias, PTCs had more follicular epithelial cells with staining for ERα and PR, however, less for ERβ. Furthermore, nodular hyperplasias had more follicular epithelial cells with staining for ERα and PR, however, less for ERβ when compared with normal thyroid tissues. As shown in Tables 2 and 3, in PTCs, high expression (≥5) was present in 109 (50%), 33 (15.1%) and 116 (53.2%) of 218 cases for ERα, ERβ and PR, respectively. High expression rates of ERα and PR were significantly higher in PTCs than in nodular hyperplasias (37.2%, P = 0.014 and 39.7%, P = 0.010 for ERα and PR, respectively) and normal thyroid tissues (6.3%, P < 0.001 and 8.6%, P < 0.001 for ERα and PR, respectively). However, high expression rate of ERβ was significantly lower in PTCs than in nodular hyperplasias (42.3%, P < 0.001) and normal thyroid tissues (56%, P < 0.001). Obviously, nodular hyperplasias had higher rates of high ERα and PR expression (P < 0.001 for both ERα and PR) and lower rate of high ERβ expression (P = 0.013) when compared with normal thyroid tissues. As for ERα36, EGFR and HER2, there were almost no follicular epithelial cells with staining for ERα36, EGFR and HER2 in normal thyroid tissues and nodular hyperplasias. However, in PTCs, there were a lot of follicular epithelial cells with staining for the three molecules. As shown in Table 2, like normal thyroid tissues, the majority of nodular hyperplasias had negative or 1 IHC score for ERα36, EGFR and HER2, no cases showed high expression (≥5) of the three molecules. However, in PTCs, the majority of cases had ≥2 IHC score for the three molecules, high expression (≥5) was present in 112 (51.4%), 132 (60.6%) and 135 (61.9%) of 218 cases for ERα36, EGFR and HER2, respectively. The differences in ERα36, EGFR and HER2 protein expression between PTCs and normal thyroid tissues as well nodular hyperplasias were statistically significant (P < 0.001) (Table 3).

**Table 2:** Immunohistochemical analysis of ERα, ERβ, PR, ERα36, EGFR and HER2 expression in 218 PTCs, 156 nodular hyperplasias and 175 normal thyroid tissues according to the scoring system.

Score	ERα			ERβ			PR			ERα36			EGFR			HER2		
	Normal	Nodular	PTCs	Normal	Nodular	PTCs	Normal	Nodular	PTCs	Normal	Nodular	PTCs	Normal	Nodular	PTCs	Normal	Nodular	PTCs
	thyroid tissues	hyper-plasias		thyroid tissues	hyper-plasias		thyroid tissues	hyper-plasias		thyroid tissues	hyper-plasias		thyroid tissues	hyper-plasias		thyroid tissues	hyper-plasia	
	*(n)*	*(n)*	*(n)*	*(n)*	*(n)*	*(n)*	*(n)*	*(n)*	*(n)*	*(n)*	*(n)*	*(n)*	*(n)*	*(n)*	*(n)*	*(n)*	*(n)*	*(n)*
0																		
Negative	99	15	6	3	4	16	95	12	4	143	103	9	146	102	5	147	105	5
+																		
1	21	11	14	7	12	33	25	14	16	28	51	14	24	50	12	26	48	10
2	16	22	16	11	19	61	14	20	21	4	2	20	5	4	15	2	3	14
3	17	27	32	27	23	47	14	22	25	0	0	31	0	0	26	0	0	25
4	11	23	41	29	32	28	12	26	36	0	0	32	0	0	28	0	0	29
++																		
6	7	19	46	35	39	17	10	33	32	0	0	32	0	0	36	0	0	39
8	3	15	32	43	20	15	5	27	43	0	0	34	0	0	38	0	0	36
+++																		
9	1	15	18	11	4	1	0	2	26	0	0	30	0	0	37	0	0	37
12	0	9	13	9	3	0	0	0	15	0	0	16	0	0	21	0	0	23

The immunohistochemical scores in PTCs, nodular hyperplasias and normal thyroid tissues were determined as the multiplication of proportion score and intensity score.

**Table 3:** Correlation of ERα, ERβ, PR, ERα36, EGFR and HER2 protein expression with clinicopathological parameters in 218 PTCs.

Characteristics	Case (*n*)	ERα			ERβ			PR			ERα36			EGFR			HER2		
		Low	High	*P* value	Low	High	*P* value	Low	High	*P* value	Low	High	*P* value	Low	High	*P* value	Low	High	*P* value
Tissue type																			
Normal thyroid tissues	175	164	11		77	98		160	15		175	0		175	0		175	0	
Nodular hyperplasias	156	98	58	<0.001^a^	90	66	0.013^a^	94	62	<0.001^a^	156	0	—	156	0	—	156	0	—
PTCs	218	109	109	<0.001^b^	185	33	<0.001^b^	102	116	<0.001^b^	106	112	<0.001^b^	86	132	<0.001^b^	83	135	<0.001^b^
				0.014^c^			<0.001^c^			0.010^c^			<0.001^c^			<0.001^c^			<0.001^c^
Classic PTCs	135	62	73	0.193	118	17	0.129	56	79	0.183	73	62	0.230	61	74	0.160	59	76	0.166
Follicular Variant of PTCs	36	17	19		26	10		18	18		15	21		12	24		11	25	
Tall Cell Variant of PTCs	26	16	10		22	4		15	11		10	16		7	19		8	18	
Oncocytic Variant of PTCs	21	14	7		19	2		13	8		8	13		6	15		5	16	
Age (years)																			
<45	60	35	25	0.129	48	12	0.217	32	28	0.233	34	26	0.143	28	32	0.179	27	33	0.194
≥45	158	74	84		137	21		70	88		72	86		58	100		56	102	
Gender																			
Male	54	22	32	0.117	43	11	0.216	21	33	0.180	30	24	0.240	26	28	0.132	25	29	0.151
Female	164	87	77		142	22		81	83		76	88		60	104		58	106	
Tumor size (cm)																			
T1 ≤ 2	85	66	19	<0.001	64	21	<0.001	55	30	<0.001	48	37	0.150	38	47	0.175	37	48	0.143
2 < T2 ≤ 4	81	34	47		69	12		36	45		37	44		33	48		32	49	
T3 > 4	52	9	43		52	0		11	41		21	31		15	37		14	38	
ETE																			
Absent	170	89	81	0.191	141	29	0.136	84	86	0.144	106	64	<0.001	86	84	<0.001	83	87	<0.001
Present	48	20	28		44	4		18	30		0	48		0	48		0	48	
LNM																			
Absent	113	63	50	0.078	92	21	0.141	58	55	0.164	86	27	<0.001	73	40	<0.001	73	40	<0.001
Present	105	46	59		93	12		44	61		20	85		13	92		10	95	
TNM stage																			
I-II	111	60	51	0.223	90	21	0.113	58	53	0.100	84	27	<0.001	74	37	<0.001	73	38	<0.001
III-IV	107	49	58		95	12		44	63		22	85		12	95		10	97	

*P*-values derived using Chi-square test to compare the expression of ERα, ERβ, PR, ERα36, EGFR and HER2 between subgroups defined by each clinicopathological parameter; ^a^stands for significant difference between normal thyroid tissues and nodular hyperplasias; ^b^stands for significant difference between PTCs and normal thyroid tissues; ^c^stands for significant difference between PTCs and nodular hyperplasias. *P* < 0.05 was considered to be statistically significant.

### Correlation of ERα, ERβ, PR, ERα36, EGFR and HER2 protein expression with clinicopathological features in PTCs

The correlation of ERα, ERβ, PR, ERα36, EGFR and HER2 protein expression with clinicopathological features was assessed by Chi-square test and summarized in Table 3. ERα and PR protein expression were positively correlated and ERβ protein expression was negatively correlated with tumor size (P < 0.001 for ERα, PR and ERβ), whereas the protein expression of them was not correlated with the other clinicopathological features analyzed. Notably, ERα36 protein expression was significantly correlated with ETE (P < 0.001), LNM (P < 0.001) and TNM stage (P < 0.001). PTCs with ETE, LNM and high TNM stage (III–IV) had higher rates of high ERα36 protein expression. However, there were no statistically significant differences in ERα36 protein expression between patients with different histologic subtype (P = 0.230), between older (≥45) and younger (<45) patients (P = 0.143), between male and female patients (P = 0.240), and between patients with large and small tumor size (P = 0.150). As for the two epidermal growth factor receptors, EGFR and HER2, no correlation was found to be present between the protein expression of the two molecules and histologic subtype, age, gender and tumor size of PTC patients (P = 0.160, 0.179, 0.132, 0.175 for EGFR and P = 0.166, 0.194, 0.151, 0.143 for HER2, respectively). However, EGFR and HER2 protein expression were significantly correlated with ETE (P < 0.001), LNM (P < 0.001) and TNM stage (P < 0.001). PTCs with ETE, LNM and high TNM stage (III-IV) had higher rates of high EGFR and HER2 protein expression than those with low TNM stage (I–II) and without ETE and LNM.

### Correlation of ERα, ERβ, PR, ERα36, EGFR and HER2 protein expression with one another in PTCs

The correlation of ERα, ERβ, PR, ERα36, EGFR and HER2 protein expression with one another in PTCs was assessed by Spearman rank test. As shown in Table 4, there was no correlation between the protein expression of ERα, ERβ or PR and the protein expression of ERα36, EGFR or HER2. However, there was some correlation between ERα, ERβ and PR protein expression, and between ERα36, EGFR and HER2 protein expression. As for ERα, ERβ and PR, there was a significant positive correlation between ERα and PR protein expression (r_s_ = 0.607, P < 0.001) and a significant negative correlation between ERα and ERβ protein expression (r_s_ = −0.294, P < 0.001) and between PR and ERβ protein expression (r_s_ = −0.245, P < 0.001). As for ERα36, EGFR and HER2, there was a significant positive correlation between ERα36 and EGFR (r_s_ = 0.285, P < 0.001), between ERα36 and HER2 (r_s_ = 0.352, P < 0.001) and between EGFR and HER2 (r_s_ = 0.160, P = 0.018) protein expression.

**Table 4:** Correlation of ERα, ERβ, PR, ERα36, EGFR and HER2 protein expression with one another in 218 PTCs.

Proteins	ERα				ERβ				PR				ERα36				EGFR			
	Low	High	*r*_*s*_	*P* value	Low	High	*r*_*s*_	*P* value	Low	High	*r*_*s*_	*P* value	Low	High	r_s_	*P* value	Low	High	*r*_*s*_	*P* value
ERβ																				
Low	81	104	−0.294	<0.001																
High	28	5																		
PR																				
Low	84	18	0.607	<0.001	77	25	−0.245	<0.001												
High	25	91			108	8														
ERα36																				
Low	57	49	0.073	0.280	86	20	−0.101	0.136	55	51	0.099	0.144								
High	52	60			99	13			47	65										
EGFR																				
Low	48	38	0.094	0.167	69	17	−0.104	0.125	44	42	0.071	0.298	57	29	0.285	<0.001				
High	61	71			116	16			58	74			49	83						
HER2																				
Low	47	36	0.104	0.126	67	16	−0.091	0.183	42	41	0.060	0.379	59	24	0.352	<0.001	41	42	0.160	0.018
High	62	73			118	17			60	75			47	88			45	90		

*P*-values for Spearman rank test; ERα, ERβ, PR, ERα36, EGFR and HER2 were tested pairwise. *P* < 0.05 was considered to be statistically significant.

### Association of concomitant high expression of ERα36, EGFR and HER2 with ETE, LNM and high TNM stage in PTCs

**Figure 2:**
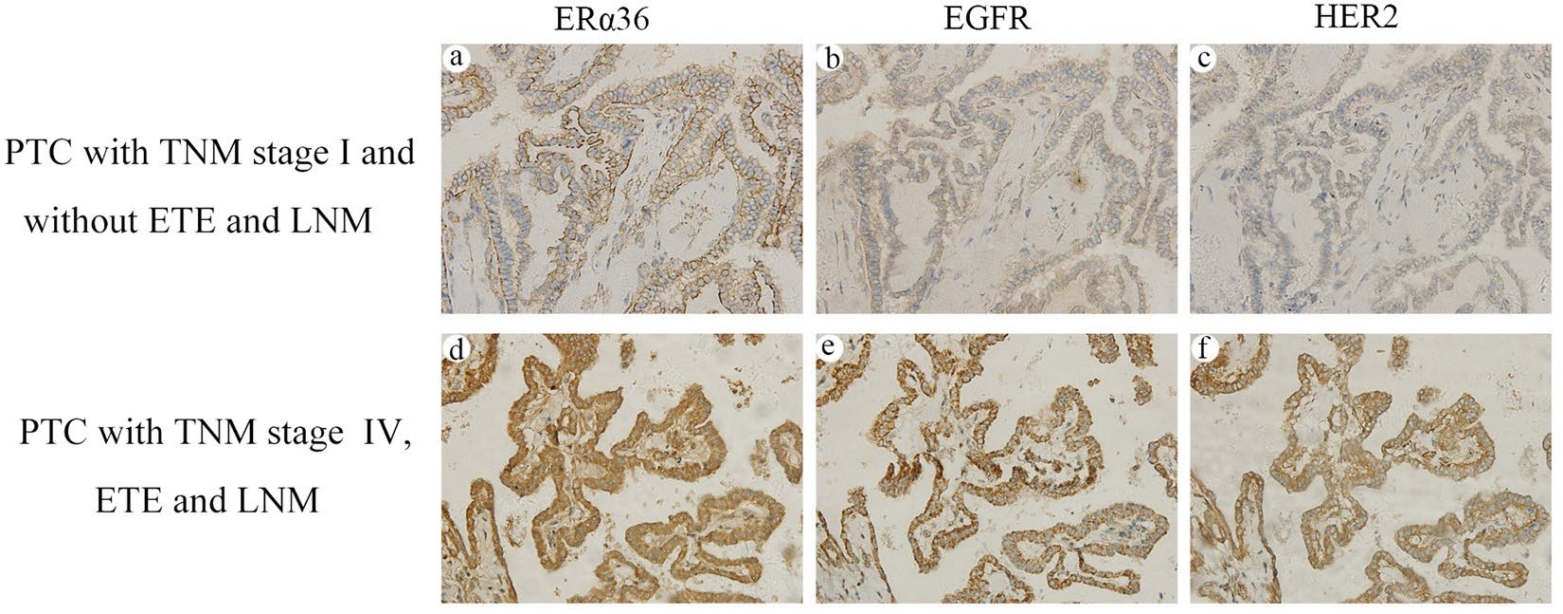
Association of concomitant high expression of ERα36, EGFR and HER2 with ETE, LNM and high TNM stage in PTCs. Columns correspond to immunostaining for ERα36, EGFR and HER2, respectively. The first row is the immunostaining of a representative of PTC with TNM stage I and without ETE and LNM showing low expression of ERα36 (**a**), EGFR (**b**) and HER2 (**c**). The second row is the immunostaining of a representative of PTC with TNM stage IV, ETE and LNM showing high expression of ERα36 (**d**), EGFR (**e**) and HER2 (**f**). All the pictures are in high-power fields (×400).

Given that ERα36, EGFR and HER2 protein expression were positively correlated with one another and statistical analysis showed that PTCs with ETE, LNM and high TNM stage had higher rates of high protein expression of the three molecules than those with low TNM stage and without ETE and LNM, we further evaluated the association of ETE, LNM and high TNM stage with concomitant high expression of ERα36, EGFR and HER2. As shown in Table 5, there were 79 PTCs showing concomitant high expression of the three molecules, including 78 with high TNM stage and 1 with low TNM stage, 77 with LNM and 2 without LNM, 48 with ETE (all of PTCs with ETE) and 31 without ETE, respectively. The incidence of high TNM stage, LNM and ETE was significantly higher in patients with ERα36 high expression combined with both EGFR and HER2 high expression (98.7% for high TNM stage, 97.5% for LNM and 63.2% for ETE, respectively) than in those patients with ERα36 high expression combined with either EGFR or HER2 high expression (50.0%, 55.6% for high TNM stage, 50.0%, 55.6% for LNM and 0% for ETE, respectively), than in those patients with only ERα36 high expression (5.0% for high TNM stage, 5.0% for LNM and 0% for ETE, respectively), and than in those patients without high expression of any of the three molecules (0% for high TNM stage, LNM and ETE). Obviously, concomitant high expression of all the three molecules was significantly associated with high TNM stage, LNM and ETE when compared with cases not showing such expression (P < 0.001). As demonstrated in Fig. 2, a-c is a representative of PTC with TNM stage I and without ETE and LNM showing low expression of ERα36, EGFR and HER2; d-f is a representative of PTC with TNM stage IV, ETE and LNM showing high expression of all the three molecules, ERα36, EGFR and HER2, respectively. When the concomitant high expression of ERα36, EGFR and HER2 was used as a predictive indicator for high TNM stage, LNM and ETE, the sensitivity, specificity, positive predictive value (PPV), negative predictive value (NPV) and diagnostic accuracy were 72.9%, 99.1%, 98.7%, 79.1%, 86.2% for high TNM stage, 73.3%, 98.2%, 97.5%, 79.9%, 86.2% for LNM and 100.0%, 81.8%, 63.2%, 100.0%, 85.8% for ETE, respectively.

**Table 5:** Correlation of concomitant expression of ERα36, EGFR and HER2 with ETE, LNM and TNM stage.

	ETE			LNM			TNM stage		
	Absent *n* (%)	Present *n* (%)	*P* value	Absent *n* (%)	Present *n* (%)	*P* value	I–II *n* (%)	III–IV *n* (%)	*P* value
Expression information									
(1) All of ERα36/EGFR/HER2 low expression	21 (100.0)	0 (0)	<0.001^a^	21 (100.0)	0 (0)	<0.001^a^	21 (100.0)	0 (0)	<0.001^a^
(2) ERα36 high expression; EGFR/HER2 low expression	20 (100.0)	0 (0)	<0.001^b^	19 (95.0)	1 (5.0)	<0.001^b^	19 (95.0)	1 (5.0)	<0.001^b^
(3) EGFR high expression; ERα36/ HER2 low expression	38 (100.0)	0 (0)	<0.001^c^	31 (81.6)	7 (18.4)	<0.001^c^	31 (81.6)	7 (18.4)	<0.001^c^
(4) HER2 high expression; ERα36/EGFR low expression	36 (100.0)	0 (0)	<0.001^d^	29 (80.6)	7 (19.4)	<0.001^d^	30 (83.3)	6 (16.7)	<0.001^d^
(5) ERα36/EGFR high expression; HER2 low expression	4 (100.0)	0 (0)	0.016^e^	2 (50.0)	2 (50.0)	0.001^e^	2 (50.0)	2 (50.0)	<0.001^e^
(6) ERα36/HER2 high expression; EGFR low expression	9 (100.0)	0 (0)	0.001^f^	4 (44.4)	5 (55.6)	<0.001^f^	4 (44.4)	5 (55.6)	<0.001^f^
(7) EGFR/HER2 high expression; ERα36 low expression	11 (100.0)	0 (0)	<0.001^g^	5 (45.5)	6 (54.5)	<0.001^g^	3 (27.3)	8 (72.7)	<0.001^g^
(8) All of ERα36/EGFR/HER2 high expression	31 (36.8)	48 (63.2)	<0.001^h^	2 (2.5)	77 (97.5)	<0.001^h^	1 (1.3)	78 (98.7)	<0.001^h^
Evaluation index **(Concomitant high expression of ERα36/EGFR/HER2)**			**Rate (%)**			**Rate (%)**			**Rate (%)**
Sensitivity			100.0			73.3			72.9
Specificity			81.8			98.2			99.1
Positive predictive value(PPV)			63.2			97.5			98.7
Negative predictive value(NPV)			100.0			79.9			79.1
Diagnostic accuracy			85.8			86.2			86.2

Correlation of concomitant expression of ERα36, EGFR and HER2 with ETE, LNM and TNM stage was measured by Chi-square test. ^a–g^Stand for significant differences between group (1)‒(7) and group (8), respectively; ^h^stands for significant difference between groups with and without concomitant high expression of all the three molecules. *P* < 0.05 was considered to be statistically significant.

## Discussion

Clinical and epidemiological studies have suggested that estrogen may be involved in the occurrence and development of PTC^1–4^, as largely demonstrated in breast, endometrial and ovarian carcinomas^5^. It is widely accepted that estrogen acts via ERα and ERβ, both of which function indisputably as hormone-dependent transcription factors to induce transactivation of a series of estrogen-dependent target genes^6,7^. PR is a paragon of estrogen-induced protein and its presence is a marker of a functional ERα^8,9^. Traditionally, ERα and PR expression are employed as biomarkers of endocrine therapy sensitivity and prognosis in breast cancer^10^. However, in the past 10 years, ERα36 (a novel 36 kDa variant of ERα) has been identified as a new member of the ER family and was found to be mainly located in the cytoplasm, as well as on the cell surface where it mediates non-genomic estrogen signaling through cross-talk with growth factor receptors and other signaling molecules (such as MAPK/ERK, PI3K/AKT and PKC) and promotes cell growth, invasion, migration and resistance to endocrine therapy^13–17^. EGFR and HER2 are two well-studied epidermal growth factor receptors and have been shown to be prognostic relevance in a variety of human malignancies including PTC^27–32^. To date, studies have shown that ERα36 is overexpressed in breast cancer stem cells^18–21^, ER-positive and -negative human breast carcinomas^22^, endometrial carcinomas^23^ and gastric carcinomas^24^, which is associated with malignancy, invasion, metastasis, drug resistance and poor prognosis of these tumors. However, no study dealt with the expression of ERα36 together with ERα, ERβ, PR, EGFR and HER2 and systematically assessed the correlation of their expression with clinicopathological features in PTC. In our present study, we simultaneously examined ERα, ERβ, PR, ERα36, EGFR and HER2 mRNA and protein expression in PTCs, nodular hyperplasias and normal thyroid tissues using real time RT-PCR and immunohistochemical staining and demonstrated that ERα, ERβ and PR mRNA and protein were expressed in PTCs, nodular hyperplasias and normal thyroid tissues. Obviously, the mRNA and protein expression of ERα and PR were gradually increased and those of ERβ were gradually decreased from normal thyroid tissues to nodular hyperplasias and to PTCs. This result is in line with the previous studies showing that increased ERα and PR expression and decreased ERβ expression are associated with the occurrence of nodular hyperplasia and PTC^33–35^. As for ERα36, EGFR and HER2, the mRNA expression levels of the three molecules were significantly higher in PTCs than in nodular hyperplasias as well as normal thyroid tissues. There were no statistically significant differences in mRNA expression of ERα36, EGFR and HER2 between nodular hyperplasias and normal thyroid tissues. Consistent with the mRNA expression of ERα36, EGFR and HER2, no cases of normal thyroid tissue and nodular hyperplasia showed high protein expression of ERα36, EGFR and HER2. However, in PTCs, high protein expression was present in 51.4%, 60.6% and 61.9% for ERα36, EGFR and HER2, respectively. The differences in ERα36, EGFR and HER2 protein expression between PTCs and normal thyroid tissues as well nodular hyperplasias were statistically significant (P  < 0.001). These results suggested that the increased mRNA and protein expression of ERα36, EGFR and HER2 are only associated with the occurrence of PTC, but not with that of nodular hyperplasia.

Then we assessed the correlation of ERα, ERβ, PR, ERα36, EGFR and HER2 protein expression with clinicopathological features. We found that ERα and PR protein expression were positively correlated and ERβ protein expression was negatively correlated with tumor size, whereas the protein expression of them was not correlated with the other clinicopathological features analyzed. These results are in line with our and other researcher’s previous studies showing that ERα and PR exert proliferative action and ERβ has an anti-proliferative function in breast cancer cells and PTC cells^36–40^. Notably, ERα36 protein expression was significantly correlated with ETE, LNM and TNM stage, whereas there was no correlation between the protein expression of ERα36 and the histologic subtype, age, gender and tumor size of PTC patients. PTCs with ETE, LNM and high TNM stage (III–IV) had higher rates of high ERα36 protein expression than those with low TNM stage (I-II) and without ETE and LNM. These results are in line with the previous studies in other tumor types such as breast, endometrial and gastric tumors^22–24^, indicating that ERα36 may also play important roles in progression and metastasis of PTC. As for EGFR and HER2, no correlation was found to be present between the protein expression of EGFR and HER2 and the histologic subtype, age, gender and tumor size of PTC patients. However, EGFR and HER2 protein expression were significantly correlated with ETE, LNM and TNM stage. PTCs with ETE, LNM and high TNM stage (III–IV) had higher rates of high EGFR and HER2 protein expression than those with low TNM stage (I–II) and without ETE and LNM. These results are consistent with the previous studies showing that EGFR and HER2 high expression are associated with some aggressive behaviors of PTC^31,32^.

Subsequently, we assessed the correlation of ERα, ERβ, PR, ERα36, EGFR and HER2 protein expression with one another. No correlation was found between the protein expression of ERα, ERβ or PR and the protein expression of ERα36, EGFR or HER2. However, we found that there was some correlation between ERα, ERβ and PR protein expression, and between ERα36, EGFR and HER2 protein expression. As for ERα, ERβ and PR, there was a significant positive correlation between ERα and PR protein expression (r_s_ = 0.607, P < 0.001) and a significant negative correlation between ERα and ERβ protein expression (r_s_ = −0.294, P < 0.001) and between PR and ERβ protein expression (r_s_ = −0.245, P < 0.001). These correlations are in line with the previous studies indicating that PR is a typical estrogen dependent target gene which is positively regulated by ERα and negatively regulated by ERβ^41,42^. Furthermore, ERα and ERβ expression levels were reversely regulated by several mechanisms such as proteasome pathway^43,44^ and some microRNAs^45,46^. As for ERα36, EGFR and HER2, there was a significant positive correlation between ERα36, EGFR and HER2 protein expression in PTCs. ERα36 expression was positively correlated with EGFR expression (r_s_ = 0.285, P < 0.001) and HER2 expression (r_s_ = 0.352, P < 0.001). Moreover, a significant positive correlation (r_s_ = 0.160, P = 0.018) was also present between EGFR and HER2 expression. The existence of these positive correlations could be supported by the following data. A positive feedback loop between ERα36 and EGFR/HER2 was reported to promote malignant growth. EGFR signaling activated transcription of ERα36 through an activator-protein-1-binding site in the promoter of ERα36. In turn, ERα36 interacted with the EGFR/Src/Shc complex to strengthen the EGFR signaling pathway and stabilize EGFR protein^17,47^. A similar positive feedback loop between ERα36 and HER2 was also reported^47,48^.

Given that ERα36, EGFR and HER2 protein expression were positively correlated with one another and the expression of these individual molecules was related to ETE, LNM and TNM stage, we subsequently evaluated the association of concomitant expression of ERα36, EGFR and HER2 with ETE, LNM and TNM stage in PTCs. The results showed that ERα36 high expression combined with both EGFR and HER2 high expression had stronger correlation with ETE, LNM and high TNM stage when compared with ERα36 high expression combined with either EGFR or HER2 high expression (P = 0.016, 0.001 for ETE, P ≤ 0.001 for LNM, P < 0.001 for high TNM stage, respectively) and only ERα36 high expression (P < 0.001 for ETE, LNM and high TNM stage). It was indicated that concomitant high expression of ERα36, EGFR and HER2 was strongly associated with ETE, LNM and high TNM stage, and may be used as a predictive indicator for malignant behaviors such as ETE, LNM and high TNM stage in PTCs.

In summary, in the present study, we simultaneously examined ERα, ERβ, PR, ERα36, EGFR and HER2 expression, systematically assessed the association of their expression with clinicopathological features and evaluated the potential usefulness of these molecules in prediction for aggressive behaviors of PTCs. The results demonstrated that the mRNA and protein expression of ERα and PR were gradually increased and those of ERβ were gradually decreased from normal thyroid tissues to nodular hyperplasias and to PTCs. Increased ERα and PR and decreased ERβ mRNA and protein expression were associated with the occurrence of nodular hyperplasia and PTC. Remarkably, the mRNA and protein expression levels of ERα36, EGFR and HER2 were significantly higher in PTCs than in nodular hyperplasias and normal thyroid tissues. There were no significant differences in the mRNA and protein expression of ERα36, EGFR and HER2 between nodular hyperplasias and normal thyroid tissues. Increased mRNA and protein expression of ERα36, EGFR and HER2 were only associated with the occurrence of PTC, but not with that of nodular hyperplasia. There was no correlation between the protein expression of ERα, ERβ or PR and the protein expression of ERα36, EGFR or HER2. However, there was some correlation between ERα, ERβ and PR protein expression, and between ERα36, EGFR and HER2 protein expression in PTCs. As for ERα, ERβ and PR, there was a significant positive correlation between ERα and PR, and a significant negative correlation between ERα and ERβ and between PR and ERβ protein expression. As for ERα36, EGFR and HER2, there was a significant positive correlation between ERα36, EGFR and HER2 protein expression in PTCs. Concomitant high expression of ERα36, EGFR and HER2 was strongly associated with aggressive behaviors including ETE, LNM and high TNM stage, and may be used as a predictive indicator for ETE, LNM and high TNM stage in PTCs.

## Materials and Methods

### Case selection and tissue sample preparation

Tumor specimens for immunohistochemical analysis were obtained from 218 PTC patients who underwent initial thyroidectomy in the Department of Surgery, the First Affiliated Hospital, Chongqing Medical University, between Jan 2010 and Jan 2015. At the initial thyroid surgery for the 218 PTC patients, cervical lymph node dissection (CLND) was performed, tumor size was assessed, histologic subtype, extrathyroidal extension (ETE) and distant metastasis were confirmed. There were 135 patients confirmed to be classic PTC, 36 patients confirmed to be follicular variant of PTC, 26 patients confirmed to be tall cell variant of PTC and 21 patients confirmed to be oncocytic variant of PTC. There were 48 patients confirmed to have ETE, 105 patients confirmed to have lymph node metastasis (LNM), 61 patients confirmed to have distant metastasis, 85 PTCs with tumor size of ≤2 cm, 81 with tumor size of >2 and ≤4 cm, 52 with tumor size of >4 cm. There were 54 men and 164 women, 60 patients with the age of <45 years and 158 with the age of ≥45 years. According to TNM classification, there were 73 patients with stage I, 38 with stage II, 18 with stage III, and 89 with stage IV. For statistical analysis, stage I and II were combined into low TNM stage (I–II), and stage III and IV were combined into high TNM stage (III–IV). Besides, benign thyroid disease specimens were obtained from 156 patients with nodular hyperplasia. 175 normal thyroid tissues were taken from the contralateral lobe of PTC specimens, which exhibited apparently normal morphology as a control. The study protocol was approved by the Ethics Committee of Chongqing Medical University and informed consent was obtained from all patients.

Tumor specimens for real-time RT-PCR were obtained from 10 PTC patients between Jan 2017 and June 2017. The benign thyroid disease specimens were obtained from 10 patients with nodular hyperplasia. For controls, 10 normal thyroid tissue specimens were used. All specimens were immediately snap-frozen in liquid nitrogen and stored at −80 °C up to subsequent RNA extraction, reverse transcription and real-time PCR.

### Tissue microarray (TMA)

Formalin-fixed, paraffin-embedded blocks were routinely prepared from surgical specimens of PTC, nodular hyperplasia and normal thyroid tissue. Representative areas containing tumor, nodular hyperplasia or normal thyroid tissue were identified by a pathologist. Duplicate tissue cores with a diameter of 0.6 mm were taken from each specimen (Beecher Instruments, Silver Springs, USA) and arrayed on a recipient paraffin block using standard procedures. Serial 5-μm-thick sections were cut with a Leica microtome (Leica Microsystems, Wetzlar, Germany) and mounted onto polylysine-coated slides.

### Immunohistochemical staining

Immunohistochemical staining of TMA section was performed as previously described^49^. Rabbit polyclonal antibodies for ERα (BS1114), ERβ (BS8465), PR (BS1766) and EGFR (BS1533) were purchased from Bioworld Technology (Minnesota, USA). Rabbit polyclonal antibody for ERα36 (CY1109) was purchased from Cell Applications (San Diego, CA, USA). Rabbit polyclonal antibody for HER2 (ab131490) was purchased from Abcam (Cambridge, MA, USA). These rabbit polyclonal antibodies were used as primary antibodies at 1:100 dilution and biotinylated goat-anti-rabbit IgG (ZB-2010, Zhongshan Golden Bridge Biotechnology, China) was used as a secondary antibody at 1:500 dilution.

### Immunohistochemical scoring

Stained TMA sections were scanned using the Nanozoomer HT Scan System (Hamamatsu Photonics, Japan). A semiquantitative assessment of immunohistochemical (IHC) scoring was performed by two observers blinded to the clinicopathologic data using the NDP Viewer software (version 1.1.27), with a consensus reached in all cases. The IHC score was assigned based on staining intensity and percentage of positive cells. The intensity score was assigned as 0 (no staining), 1 (weak staining), 2 (moderate staining) and 3 (strong staining). The proportion score was assigned as 0 (<5% positive cells), 1 (6–25% positive cells), 2 (26–50% positive cells), 3 (51–75% positive cells) and 4 (>75% positive cells). Multiplication of the intensity and proportion scores gave rise to the final staining score: 0 (negative), + (1–4), ++ (5–8) and +++ (9–12). For statistical analysis, a final staining score of negative or + was defined as the low expression, and a final staining score of +  + or +++ was defined as the high expression.

### RNA Extraction, Reverse Transcription and Real-Time PCR

Total RNA was extracted from frozen PTC, nodular hyperplasia and normal thyroid tissue specimens using TRIzol reagent (Invitrogen, Camarillo, CA, USA), and residual genomic DNA was eliminated by DNase I digestion (Ambion, USA). RNA purity was confirmed by spectrophotometry. Total RNA was reverse transcribed into cDNA by using SuperScript III Reverse Transcriptase (Invitrogen, USA) according to the manufacturer’s protocol. The final cDNA product was amounted to 25 μL and stored at −80 °C.

Real-time PCR was performed by using SYBR-Green real-time PCR method on ABI-Prism 7000 sequence detector (Applied Biosystems, USA). The primers used were as following: for ERα, 5′-ATGATGAAAGGTGGGATACGA3′ (forward) and 5′-CTAGTTTGCGAGATTCTTCTTGTC-3′ (reverse); for ERβ, 5′-GTCACAGCGACCCAGGAT-3′ (forward) and 5′-CTTACTTCTACCTCTGAGAAAAC-3′ (reverse); for PR, 5′-TCATTCTATTCATTATGCCTTACCA-3′ (forward) and 5′-GAAAACCTTCCCGATGCTTCAG-3′ (reverse); for ERα36, 5′-CCAAGAATGTTCAACCACAACCT-3′ (forward) and 5′-GCACGGTTCATTA ACATCTTTCTG-3′ (reverse); for EGFR, 5′-CGTCCGCAAGTGTAAGAA-3′(forward) and 5′-AGCAAA AACCCTGTGATT-3′ (reverse); for HER2, 5′-AGGGAGTATGTGAATGCC-3′(forward) and 5′-GGCCACTG GAATTTTCAC-3′(reverse); for GAPDH, 5′-GAAGGTGAAGGTCGGAGT-3′ (forward) and 5′-GAAGATGGT GATGGGATTTC-3′ (reverse). Quantities of gene specific mRNA expression were determined by the CT method. Samples were analyzed in triplicate. Average threshold cycle (CT) value for GAPDH was used as an internal calibrator. The 2^−ΔΔCT^ method was used for relative quantitation. Data was presented as the mean ± standard deviation of three independent experiments. The real-time PCR mix was made on the basis of the prescription from the supplier: 6 μL sterile water, 1 μL sense and 1 μL antisense primers, 10 μL Platinum SYBR Green qPCR SuperMix-UDG w/ROX (Invitrogen, USA), and 2 μL target cDNA in a total volume of 20 μL. Run conditions were 50 °C for 2 min, 95 °C for 10 min, followed by 40 cycles at 95 °C for 15 s and 60 °C for 1 min.

### Statistical analysis

Statistical analysis was performed using SPSS 18.0 statistical software. Data were presented as percentages and mean and standard deviation, according to the distribution. Significance was assessed using Chi-square, Spearman rank and Mann-Whitney U tests as appropriate, to compare the groups. P value < 0.05 was considered to be statistically significant.

### Data Availability

The data generated or analysed during this study are included in this published article and its Supplementary Information files.
